# Utilization of Child-Appropriate Medicines: Use of Pediatric Use Marketing Authorisation (PUMA) Products in Croatia

**DOI:** 10.3390/jcm14175994

**Published:** 2025-08-25

**Authors:** Andrej Belančić, Marta Kučan Štiglić, Arnes Rešić, Almir Fajkić, Dinko Vitezić

**Affiliations:** 1 Department of Basic and Clinical Pharmacology and Toxicology, Faculty of Medicine, University of Rijeka, 51000 Rijeka, Croatia; kucanmarta@gmail.com (M.K.Š.); dvitezic@gmail.com (D.V.); 2 Children’s Hospital Zagreb, 10000 Zagreb, Croatia; aresic2@gmail.com; 3 Faculty of Health Sciences, University of Split, 21000 Split, Croatia; 4 Department of Pathophysiology, University of Sarajevo, 71000 Sarajevo, Bosnia and Herzegovina; almir.fajkic@mf.unsa.ba

**Keywords:** drug utilization, off-label use, pediatrics, pediatric use marketing authorisation, pharmacoepidemiology

## Abstract

**Background/Objectives**: Children remain underserved in pharmaceutical development, with off-label prescribing still prevalent in part due to a lack of age-appropriate formulations. This study aimed to evaluate the national uptake of Pediatric Use Marketing Authorisation (PUMA)-labelled medicines in Croatia from 2017 to 2024. **Methods**: We conducted a retrospective, descriptive pharmacoepidemiological study using the IMS (Intercontinental Medical Statistics) and IQVIA (Information, Quintiles, VIA; formerly IMS Health and Quintiles) datasets to track utilization and the expenditure of all PUMA products. Utilization was assessed using defined daily doses per 1000 inhabitants per day (DDDs/1000/day) and annual product dispensation counts. **Results**: Over the study period, five PUMA medicines entered the Croatian market, with usage rising from 853 packages in 2018 to 9232 in 2024. The DDDs/1000/day increased 33.8-fold, while the expenditure escalated nearly 5.8-fold, from EUR 145,898 to EUR 844,145. Midazolam and melatonin were the most frequently prescribed, yet the overall utilization remained marginal relative to pediatric needs. **Conclusions**: In conclusion, while regulatory availability of PUMA products has improved, their clinical adoption in Croatia remains limited. Addressing economic, educational, and policy barriers is essential to close the gap between authorization and utilization.

## 1. Introduction

Pediatric patients remain one of the most underserved populations in pharmaceutical development. Despite growing awareness of the risks, the off-label use of medicines—often lacking validated dosing, age-appropriate formulations, or adequate safety data—persists across pediatric care [1]. In the United States, off-label prescribing rates in pediatric settings range from 62% to 85%, with the highest rates observed in neonates and adolescents [2]. This practice is in part driven by the limited availability of medicines specifically labelled for pediatric use and the unique physiological differences between children and adults, which complicate direct extrapolation of adult data to pediatric populations [3]. This is not just a medical gap but has also been a historical structural gap in drug development policy. Regulatory efforts such as the US Pediatric Research Equity Act and the Best Pharmaceuticals for Children Act have incentivized pediatric medicine studies, resulting in some progress, but delays and incomplete compliance persist, leaving many medicines without adequate pediatric labelling even years after their approval [4]. In response, the European Union has introduced the Pediatric Use Marketing Authorisation (PUMA) under Regulation (EC) No. 1901/2006. This mechanism aims to stimulate the development of child-appropriate medicines, particularly for off-patent products, by granting 10 years of data and market exclusivity for pediatric indications, even when the active substance itself is no longer under patent protection. The incentive is designed to offset the lack of commercial potential of pediatric formulations and the studies that pharmaceutical companies need to invest in by fulfilling all regulatory processes. Obtaining a PUMA requires the creation of an agreed Pediatric Investigation Plan (PIP) with the European Medicines Agency’s Pediatric Committee (PDCO), ensuring development and evaluation specifically for the needs of children [5,6,7].

However, the real-world impact of this regulatory pathway remains limited. Only a small number of medicines have been authorized under PUMA (Table 1), and even fewer have seen widespread clinical adoption. Major challenges include limited scopes of indication, limited market access, cost-related disincentives, and inadequate alignment with local prescribing practices.

Recent analysis has revealed significant variation in the gap in access to PUMA products by European countries, primarily attributable not only to procurement and reimbursement policies but also long-entrenched off-label prescribing practices, institutional inertia, and an inadequate prescriber information drive [15]. In the United Kingdom, for example, several PUMA-authorized products are available, yet local formularies often continue to favour off-label alternatives due to cost considerations, limited indications, or an absence of national prescribing guidance. Access barriers include the five As: authorization, availability, affordability, appropriateness, and acceptability [16].

This situation highlights a central paradox: regulatory approval alone does not ensure clinical adoption. Without coordinated efforts spanning pricing, procurement, and prescriber education, the PUMA framework risks falling short of its goal—securing safe, effective, and equitable treatment options for pediatric populations.

To date, Croatia has not been systematically assessed in this regard. The present study aims to map the availability, accessibility, and integration of PUMA-authorized medicines within the Croatian healthcare system. In doing so, it indirectly speculates on key drivers—and deterrents—of pediatric-appropriate prescribing in real-world settings.

## 2. Materials and Methods

We conducted a retrospective, descriptive pharmacoepidemiological study to analyze the national utilization trends of medicines granted PUMA in Croatia between 2017 and 2024. The study examined patterns of pharmaceutical utilization and expenditure using internationally accepted metrics to facilitate temporal and cross-national comparisons.

Pharmaceutical utilization data were obtained from the International Medical Statistics (IMS) and IQVIA (Information, Quintiles, VIA; formerly IMS Health and Quintiles) databases, both of which are widely recognized sources for drug market surveillance and pharmacoepidemiological research. These databases provided data on the sales and distribution of all PUMA-labelled products marketed in Croatia during the study period.

Population data for calculating utilization rates were sourced from the Croatian Bureau of Statistics. For 2021, official census counts were used, while inter-census population estimates were applied for all other years. However, data specific to the pediatric population aged 0–18 years are not available in the Croatian national statistical system. Instead, only aggregated figures for the 0–14 and 15–24 age groups are provided. As a result, it was not feasible to calculate age-specific utilization rates (e.g., DDDs/1000 children/day) [17].

To address the absence of an accurate pediatric denominator, drug utilization was quantified using the defined daily doses per 1000 inhabitants per day (DDDs/1000/day), in accordance with the methodology of the WHO Collaborating Centre for Drug Statistics Methodology. Although the DDD system is based on adult dosing and no official pediatric DDDs exist, this metric remains the most widely used standard in pediatric drug utilization studies for assessing the relative drug use over time and across countries [18].

In order to mitigate this methodological limitation, we also reported the annual count of individual PUMA products (per unique formulation) dispensed during the study period. This product count metric, when divided by 365, may approximate daily prescribing patterns and offers an alternative, complementary measure of utilization that does not rely on population denominators.

The Anatomical Therapeutic Chemical (ATC)/DDD classification system was applied to ensure standardized assessment and facilitate cross-national comparability. In cases where the WHO revised the DDD values for specific substances during the study period, the most recent values (as of 2024) were applied retrospectively to all years to maintain methodological consistency [18].

The annual pharmaceutical expenditure for PUMA products was reported in euros (EUR). The expenditure was assessed both as the absolute annual spending and as the unit costs, expressed in the euros per DDD (EUR/DDDs) for each agent. Price data were sourced from publicly available IQVIA listings and did not account for undisclosed rebates or procurement discounts.

Temporal trends in the utilization and expenditure were assessed using an index of change, defined as the relative difference between the baseline year (2018) and the most recent year (2024). This index was calculated for both the DDDs/1000/day and absolute expenditure (EUR). All data analyses were descriptive and performed using Microsoft Excel.

## 3. Results

The use of medicines authorized under the Pediatric Use Marketing Authorisation (PUMA) scheme increased substantially over the observed period, both in terms of the volume and expenditure.

An analysis of the prescribing patterns for PUMA-authorized medicines revealed that propranolol (Hemangiol^®^) became available in 2018, followed by melatonin (Slenyto^®^) in 2021, midazolam (Buccolam^®^) and hydrocortisone (Alkindi^®^) in 2022, and glycopyrronium bromide (Sialanar^®^) in 2023.

**Figure 1:**
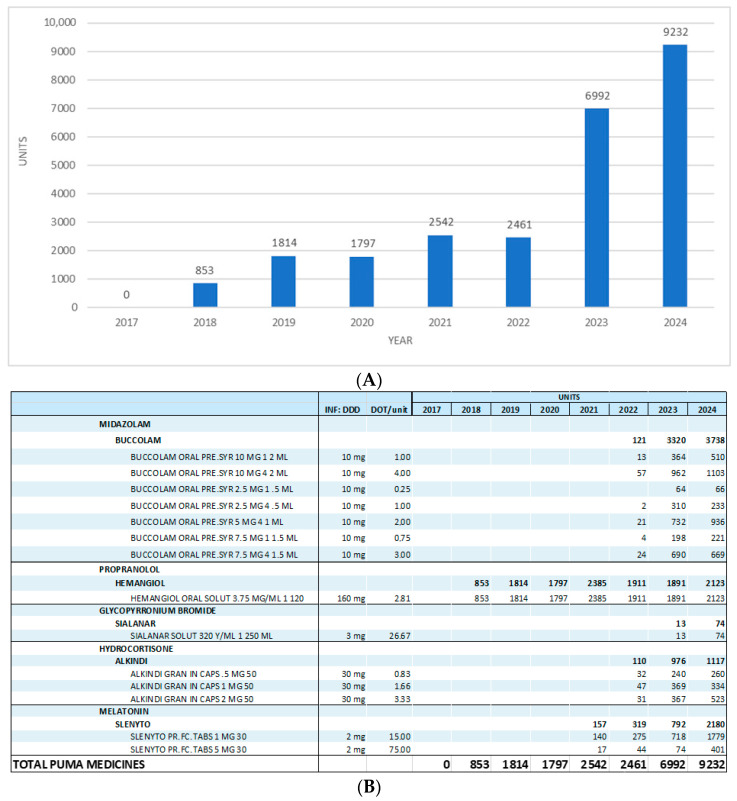
(**A**,**B**) The total use of PUMA medicines from 2017 to 2024, in units/packages. Note: Kigabeq^®^ (vigabatrin, PUMA formulation) was not available on the Croatian market and was not dispensed during the study period.

In 2018, a total of 853 packages of all the listed PUMA-authorized medicines were dispensed. A consistent upward trend was demonstrated, reaching 9232 packages in 2024 (Figure 1A,B).

**Figure 2:**
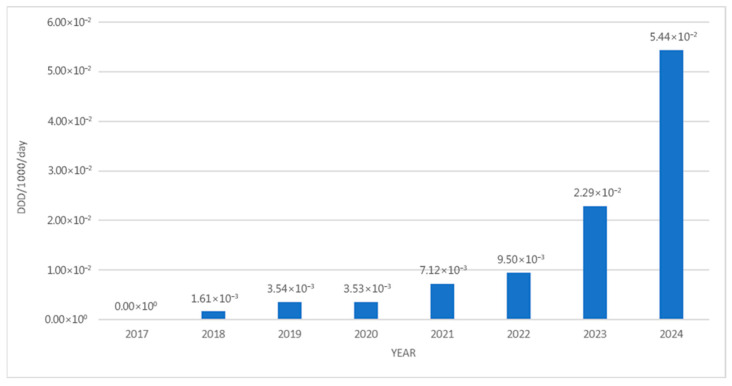
The total use of PUMA medicines from 2017 to 2024, in DDDs/1000/day.

In 2018, the total consumption amounted to 1.61 × 10^−3^ defined daily doses per 1000 inhabitants per day (DDDs/1000/day). By 2024, this value had increased approximately 33.8-fold, reaching 5.44 × 10^−2^ DDDs/1000/day (Figure 2).

**Figure 3:**
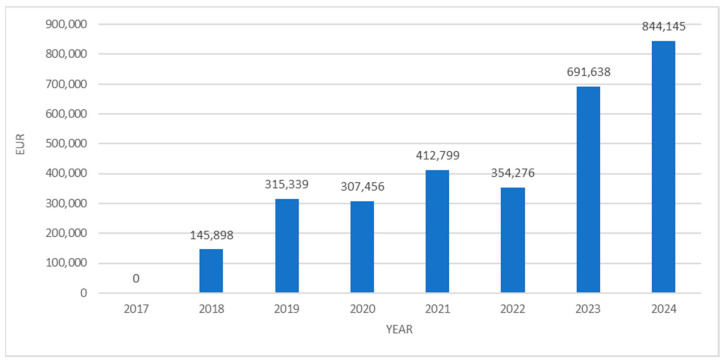
Financial expenditure on PUMA medicines from 2017 to 2024.

When analyzed in financial terms, the expenditure on these medicines rose from EUR 145,898 in 2018 to EUR 844,145 in 2024, representing nearly a 5.8-fold increase (Figure 3).

**Figure 4:**
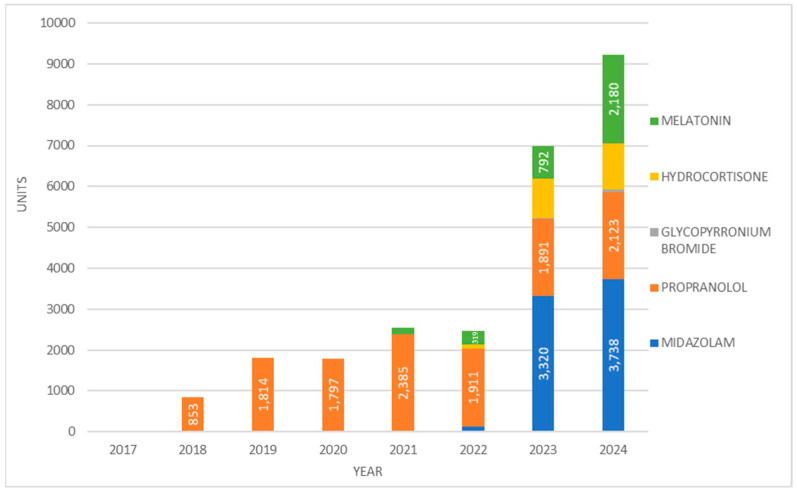
Prescription of different PUMA medicines in units/packages from 2017 to 2024.

In 2024, midazolam was the most prescribed PUMA medicine in terms of the number of dispensed packages (across all available strengths), totalling 3738, followed by melatonin (2180 packages) and propranolol (2123 packages) (Figure 4).

**Figure 5:**
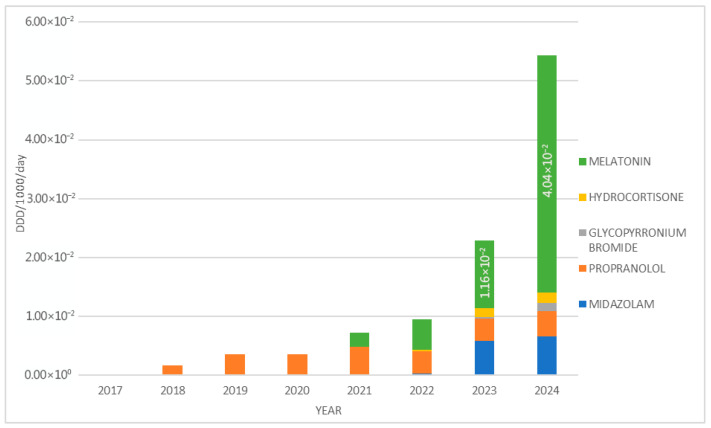
Prescription of different PUMA medicines in DDDs/1000/day from 2017 to 2024.

When measured in DDDs/1000/day, melatonin was the most prescribed PUMA medicine in 2024 (4.04 × 10^−2^), followed by midazolam (6.56 × 10^−3^) (Figure 5).

**Figure 6:**
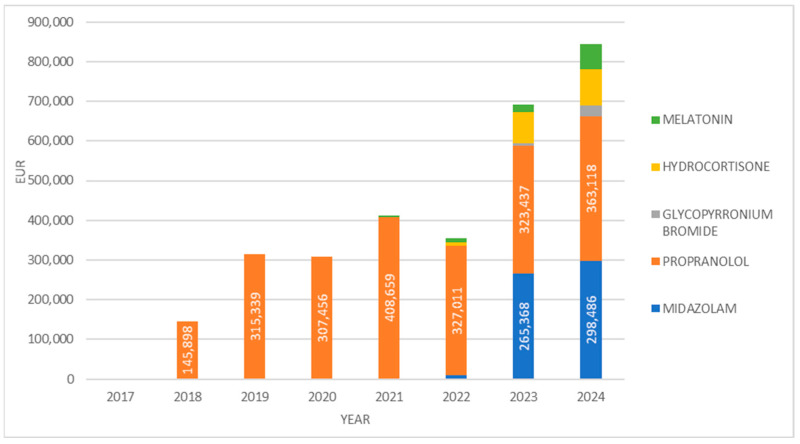
Financial expenditure on different PUMA medicines from 2017 to 2024.

When analyzed in financial terms over the entire period since its market introduction, propranolol accounted for the highest cumulative expenditure among the PUMA-authorized medicines. The annual spending on propranolol ranged from EUR 145,898 in 2018 to a peak of EUR 408,659 in 2021, followed by a slight decrease to EUR 363,118 in 2024. Midazolam ranked second in terms of financial expenditure, with EUR 298,498 spent in 2024 alone (Figure 6).

The data indicate that the average price of PUMA-authorized medicines remained relatively stable throughout the observed period. When expressed as the unit cost in EUR/DDD, propranolol had the highest price in 2024, amounting to 60.87 EUR/DDD. It was followed by hydrocortisone (36.40 EUR/DDD) and midazolam (32.39 EUR/DDD).

**Figure 7:**
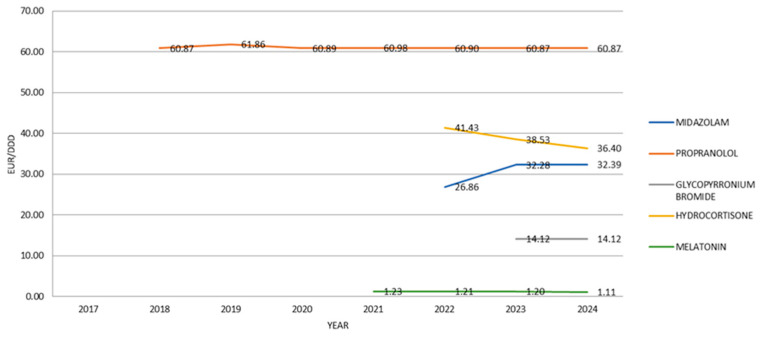
Price per 1 DDD of different PUMA medicines from 2017 to 2024.

Medicines with lower unit costs included glycopyrronium bromide (14.12 EUR/DDD), and melatonin, which had the lowest cost at 1.11 EUR/DDD (Figure 7).

## 4. Discussion

This study presents, to the best of our knowledge, one of the first pharmacoepidemiological evaluations of medicines granted PUMA in the published literature, and the first of its kind conducted in Central and Eastern Europe [19]. It provides a comprehensive national-level analysis of the utilization and expenditure trends for PUMA products in Croatia over an eight-year period (2017–2024), filling an important evidence gap in pediatric medicine policy and access monitoring.

Between 2017 and 2024, Croatia witnessed an impressive surge of medicines with PUMA that were readily accessible and consumed. In absolute terms, this upward trend is visible with 9232 packages being dispensed in 2024 compared to 853 in 2018. However, from a pharmacoepidemiological perspective, it has to be noted that the absolute number tells only half the reality. On a population basis, the total consumption barely reached 0.0544 DDDs/1000/day, which could be considered as marginal when considered against the factual background of pediatric pharmacotherapy. Thus, although these medicines’ availability expanded substantially (from one medicine in 2018 to six in 2024) and theur overall volume increased 33.8-fold, their relative penetration into everyday pediatric prescribing was still shallow, indicating that systemic, educational, or reimbursement barriers continue to limit broader clinical uptake.

Similar trends play out across the rest of the European Union. Recent research coming out of the U.K. proves that simply authorizing PUMA products does not automatically mean they get integrated into clinical practice [16]. Local cost containment policies, familiarity with off-label prescribing, and the presence of a mandatory substitution guideline all slow their adoption. A ten-year report from the European Commission similarly noted that by 2022, just six PUMA products had even been authorized, and there were disparities in the market availability among member states. Our findings echo this trend: Croatia is not lagging behind—it is simply following a relatively flawed model.

Quantifying medicine use in pediatrics remains technically problematic. The DDD, while a cornerstone of pharmacoepidemiological research, has not been formally established for pediatric populations. The WHO Collaborating Centre has not issued pediatric-specific DDD values, leading to the routine use of adult DDDs in child-oriented studies. This limitation is widely acknowledged but persists due to the lack of standardized alternatives [20,21].

As discussed, in this study utilization was calculated using the DDDs/1000/day, a WHO-endorsed metric enabling temporal and international comparability. Recognizing its limitations, we also included the annual count of the dispensed units/packages per PUMA product as an auxiliary indicator. This dual-metric approach allowed for trend analysis that was less vulnerable to demographic distortion or age-weighted dose variability—issues frequently encountered when working with aggregated pediatric data in countries like Croatia, where detailed age-stratified denominators are unavailable.

This methodological compromise, using adult-based DDDs with a general population denominator, may introduce imprecision but reflects a pragmatic and validated solution in the absence of finer-resolution data. Until harmonized pediatric-specific drug utilization metrics are established at a supranational level, this remains the most robust approach for assessing national-level access patterns [22].

The observed prescribing pattern reflects late and selective adoption. An oral propranolol solution (Hemangiol^®^) was the only PUMA-labelled product available in 2018, whilst more clinically essential products such as midazolam and hydrocortisone did not enter the market until 2022 (Figure 1B). This delay was not driven by clinical irrelevance, but by systemic inaction. Even in 2024, the most frequently dispensed medicine by volume—midazolam—reached only 3738 packages.

Melatonin, although low in cost (1.11 EUR/DDD), emerged as the most used drug when adjusted for population exposure (DDDs/1000/day). By contrast, propranolol accounted for the highest cumulative expenditure, despite relatively low usage. This disconnect between economic allocation and clinical reach is structurally problematic.

Our findings support the broader assertion that prescribing practices in pediatrics are governed less by regulation than by habit and procurement logic. Off-label options continue to prevail not because of any clinical advantage but simply because PUMA-labelled medicines are more expensive, less known, and seldom included in any clinical guidelines or institutional protocols as a requirement [16]. In addition, educational outreach is not adequate and the inclusion of such products in formularies is inconsistent. Without supportive policies, PUMA products have to compete in the market [6].

Numerous studies have proved the high level of off-label prescribing in pediatric care. International data corroborate these findings. In Australian pediatric inpatient settings, more than half (54%) of medicines were prescribed off-label, with certain medications (e.g., oxycodone, midazolam) always used off-label [23]. European and global reviews indicate that at least one-third of hospitalized children and up to 90% of neonates in intensive care units receive off-label prescriptions [24]. A study from Italy reports off-label prescribing rates of 50%, with every patient in some cohorts receiving at least one off-label medicine [25]. Lastly, as reported by Allen et al., between 3.2% and 95% of all pediatric prescriptions were off-label, with an average prevalence approximating 38% in real-world outpatient settings [26]. This demonstrates a systemic reliance on non-authorized medications, not because they are more suitable, but rather due to the unavailability of child-specific alternatives or the lack of familiarity among practitioners and economic disincentives to use them. In this environment, the uptake of PUMA-labelled medicines faces resistance rooted in inertia rather than evidence.

An often-overlooked dimension is the ethical implication of continued reliance on off-label prescribing in children. While such practice is clinically widespread and some-times unavoidable, it exposes pediatric patients to therapeutic uncertainty that would not be tolerated in adult populations. PUMA was explicitly designed to address this inequity by incentivizing manufacturers to generate child-specific data, yet our findings show that the intended benefits remain only partially realized [27,28]. From a clinical standpoint, the paradox is evident: medicines developed and authorized with pediatric safety and efficacy data are available, yet they are not systematically adopted, leaving children reliant on less rigorously validated alternatives. This situation underscores a gap not only in policy implementation but also in the ethical responsibility of health systems to ensure that children receive therapies aligned with the best available evidence [16].

Croatia’s experience with PUMA-labelled medicines reflects incremental progress constrained by structural inertia. Authorization has increased, but PUMA-labelled medicines’ integration into practice remains limited. The policy intent behind the PUMA initiative—to bridge the gap between regulatory approval and safe, age-appropriate pediatric therapy—has not yet been realized at the level of everyday prescribing. Without systemic alignment between regulation, reimbursement, and clinical training, PUMA will remain underutilized, not because it is flawed, but because health systems are not designed to support it.

### Strengths and Limitations

A major strength of this study is the use of high-quality, comprehensive data sources, the IMS and IQVIA databases, which capture aggregated medicine sales from all the wholesalers supplying both public and hospital pharmacies nationwide. These datasets ensure near-complete coverage of the pharmaceutical market and support robust assessments of national trends over time.

Utilization was measured using the WHO-recommended metric of the defined daily doses per 1000 inhabitants per day (DDDs/1000/day), enabling international comparability. Recognizing the limitations of the DDD system, particularly its basis in adult dosing and the lack of official pediatric DDDs, we also reported annual counts of the individual PUMA product formulations dispensed. This alternative measure offered a proxy for the daily prescribing activity and helped contextualize the utilization trends independently of dosing assumptions or population denominators.

However, several limitations must be acknowledged. First, the data reflect the volume of medicines distributed to pharmacies rather than prescriptions written or drugs dispensed at the patient level. Therefore, the actual medicine consumption may have been under- or overestimated, especially as stocking fluctuations within pharmacies were not recorded, although these are assumed to be relatively stable over time.

Second, official population data specific to children aged 0–18 years are not available in Croatia. Only data for age brackets of 0–14 and 15–24 years are reported by the national statistical authority, which precluded accurate calculation of pediatric-specific utilization metrics (e.g., DDDs/1000 children/day). As a result, the DDDs/1000/day was calculated using the total population as the denominator, introducing some degree of imprecision when interpreting the use among pediatric subgroups.

Finally, the absence of patient-level data limited our ability to assess the clinical appropriateness, adherence, or outcomes. Likewise, while an increase in PUMA product utilization was observed, causality (particularly regarding improved access) could not be established. Nonetheless, Croatia’s universal health coverage and centralized reimbursement system reduce the likelihood that systemic barriers to medicine access significantly confounded the findings.

## 5. Conclusions

Despite its intent to address the unmet needs of children, the PUMA framework re-mains underutilized in clinical reality. This study illustrates that while regulatory structures can stimulate availability, proper access depends on harmonized efforts across health policy, prescriber behaviour, and economic incentives. For Croatia, and indeed for the EU, bridging the gap between authorization and utilization will require more than regulatory reform; it will demand a shift in institutional priorities, clinical education, and procurement logic. Ultimately, aligning these elements is essential not only to reduce the reliance on off-label prescribing but also to safeguard equity, safety, and therapeutic justice in pediatric care.

## Figures and Tables

**Table 1:** Drugs authorized under PUMA with approved indications.

Medicine	Approved Indication/Use	Company	Year PUMA Was Obtained	References
**Buccolam** **(midazolam)**	The treatment of prolonged, acute, convulsive seizures in children aged 3 months to <18 years.	Shire	2011	[8,9]
**Hemangiol** **(oral propranolol solution)**	Proliferating infantile hemangioma requiring systemic therapy in infants aged 5 weeks to 5 months.	Pierre Fabre	2014	[10]
**Sialanar** **(oral glycopyrronium bromide solution)**	Severe sialorrhea (drooling) in children and adolescents with neurodisabilities (typically 3–17 years).	(Proveca)	2016	[11]
**Alkindi** **(hydrocortisone granules)**	Replacement therapy in pediatric patients with adrenocortical insufficiency (e.g., congenital adrenal hyperplasia, Addison’s disease).	Diurnal	2018	[12]
**Kigabeq (vigabatrin)**	West syndrome (infantile spasms) and refractory complex partial seizures in children and adolescents aged 1 month to 18 years.	Lundbeck	2018	[13]
**Slenyto** **(prolonged-release melatonin)**	Insomnia in children and adolescents aged 2–18 years with autism spectrum disorder and/or Smith–Magenis syndrome.	Neurim	2018	[14]

## Data Availability

Available upon reasonable request from the corresponding author.
